# Supplementary material for: Differential angiogenesis of bone and muscle endothelium in aging and inflammatory processes
Source: Commun Biol. 2023 Jan 31;6:126. doi: 10.1038/s42003-023-04515-9 (PMC9889796; doi:10.1038/s42003-023-04515-9)
Supplement: Supplementary file 2 — Supplementary information [file 42003_2023_4515_MOESM2_ESM.pdf]

Supplementary information

Differential angiogenesis of bone and muscle endothelium in aging and inflammatory processes

Chiara Arrigoni[1,2,3], Paola Ostano[4], Simone Bersini[1,2,3], Martina Crippa[1,2,5], Maria Vittoria Colombo[1,2,5], Mara Gilardi[6], Luigi Zagra[7], Maurizia Mello-Grand[4], Ilaria Gregnanin[4], Carmen Ghilardi[8], Maria Rosa Bani[8], Christian Candrian[2,3], Giovanna Chiorino[4], Matteo Moretti[1,2,3,7]

[1] Regenerative Medicine Technologies Lab, Laboratories for Translational Research, Ente Ospedaliero Cantonale (EOC), via F Chiesa, 5, 6500 Bellinzona, Switzerland

[2] Servizio di Ortopedia e Traumatologia, Ente Ospedaliero Cantonale, Lugano, Switzerland

[3] Euler Institute, Faculty of Biomedical Sciences, Università della Svizzera Italiana, Lugano, Switzerland

[4] Lab of Cancer Genomics, Fondazione “Edo ed Elvo Tempia”, Biella, Italy

[5] Laboratory of Biological Structures Mechanics-Chemistry, Material and Chemical Engineering Department “Giulio Natta”, Politecnico di Milano, Milan, Italy

[6] NOMIS Center for Immunobiology and Microbial Pathogenesis, Salk Institute, San Diego, CA, USA

[7] IRCCS Istituto Ortopedico Galeazzi, Cell and Tissue Engineering Laboratory, Milan, Italy

[8]Laboratory of Cancer Metastasis Therapeutics, Istituto di Ricerche Farmacologiche Mario Negri-IRCCS, Milano, Italy

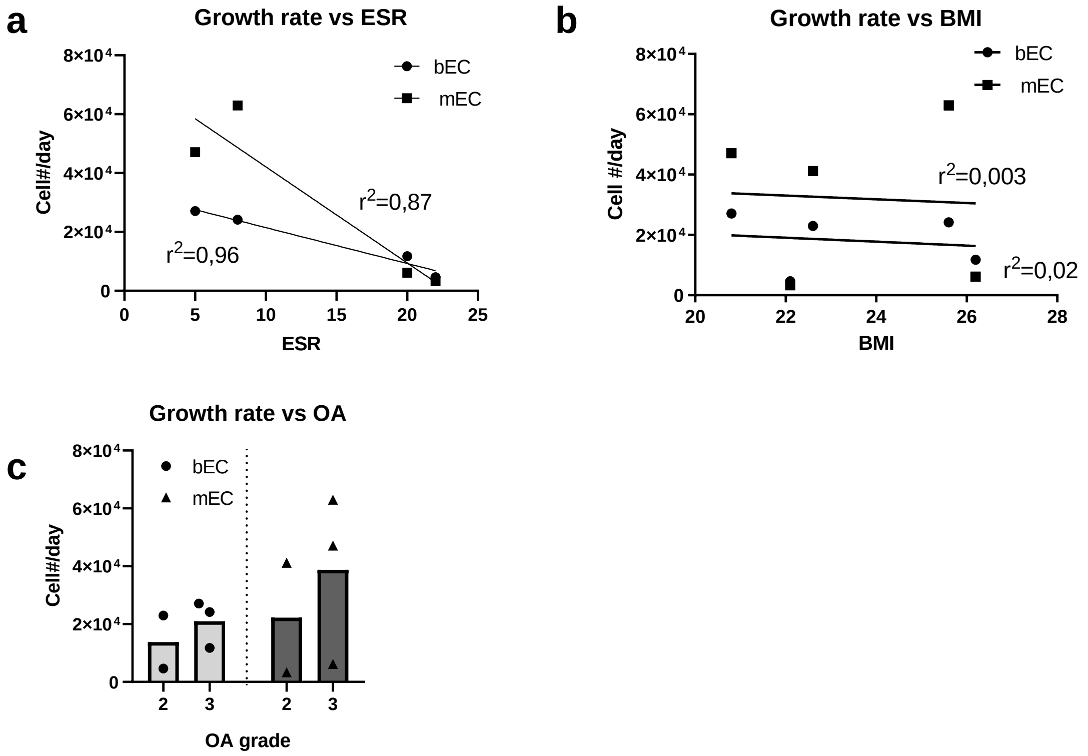

Supplementary Figure 1: A) graph showing the correlation between bEC and mEc growth rates and ESR values (n=4, p=0.017 and p=0.067 for bECs and mECs respectively). B) graph showing the correlation between bEC and mEc growth rates and BMI values (n=5, p=0.79 and p=0.93 for bECs and mECs respectively). C) graph showing bEC and mEc growth rates for different OA grades (average, n=2 for OA grade 2 and n=3 for OA grade 3).

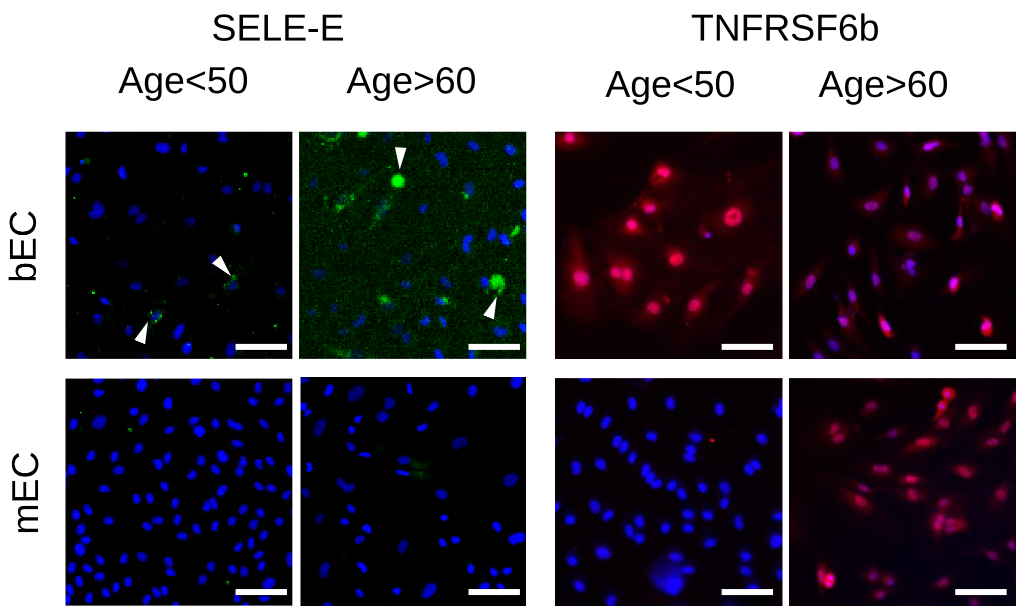

Supplementary Figure 2: Immunofluorescence staining of bECs and mECs from patients in different age ranges. Green staining: SELE-E, red staining: TNFRSF6b, nuclei in blue (DAPI). Scale bars: 50 µm.

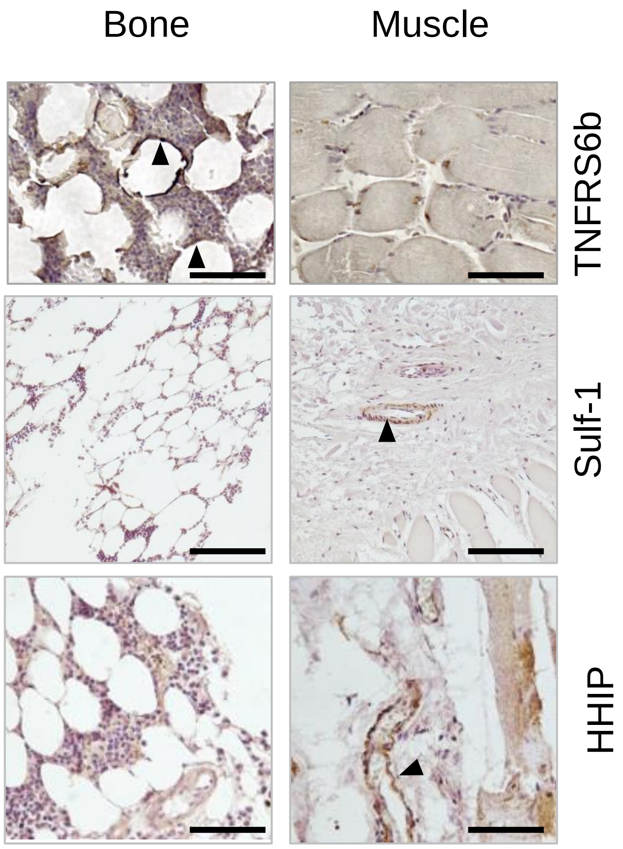

Supplementary Figure 3: Immunohistochemical staining of patient-matched bone and muscle tissues, showing higher expression of TNFRS6b in bone and higher expression of SULF-1 and HHIP in muscle, according to differential expression of encoding genes. Scale bars: 100 μm

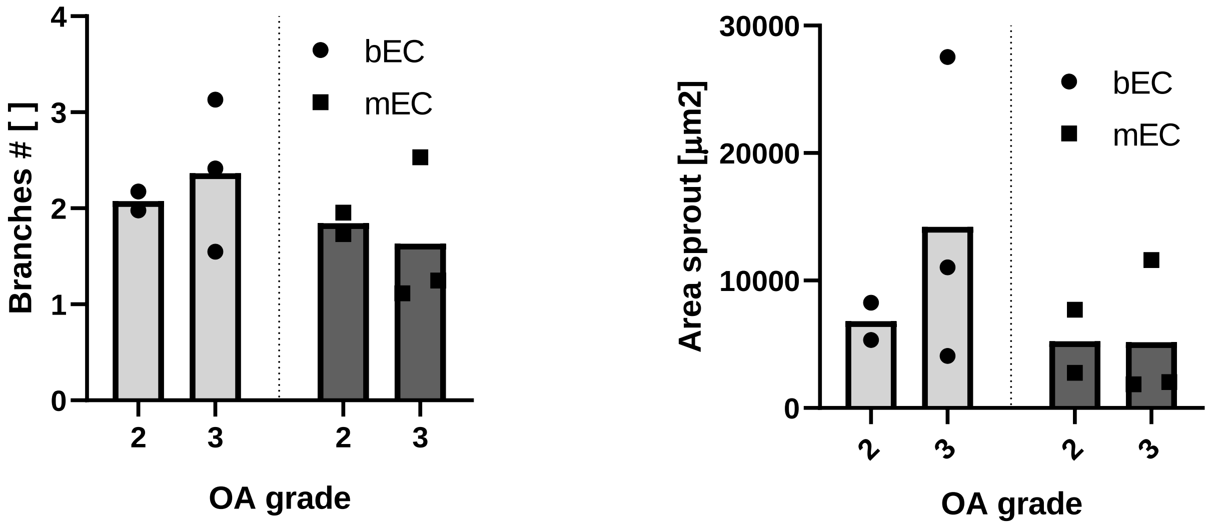

Supplementary Figure 4: comparison of branch number and area sprout (reported as average and single points) between bECs and mECs derived from patients with grade 2 (n=2) or grade 3 OA (n=3).

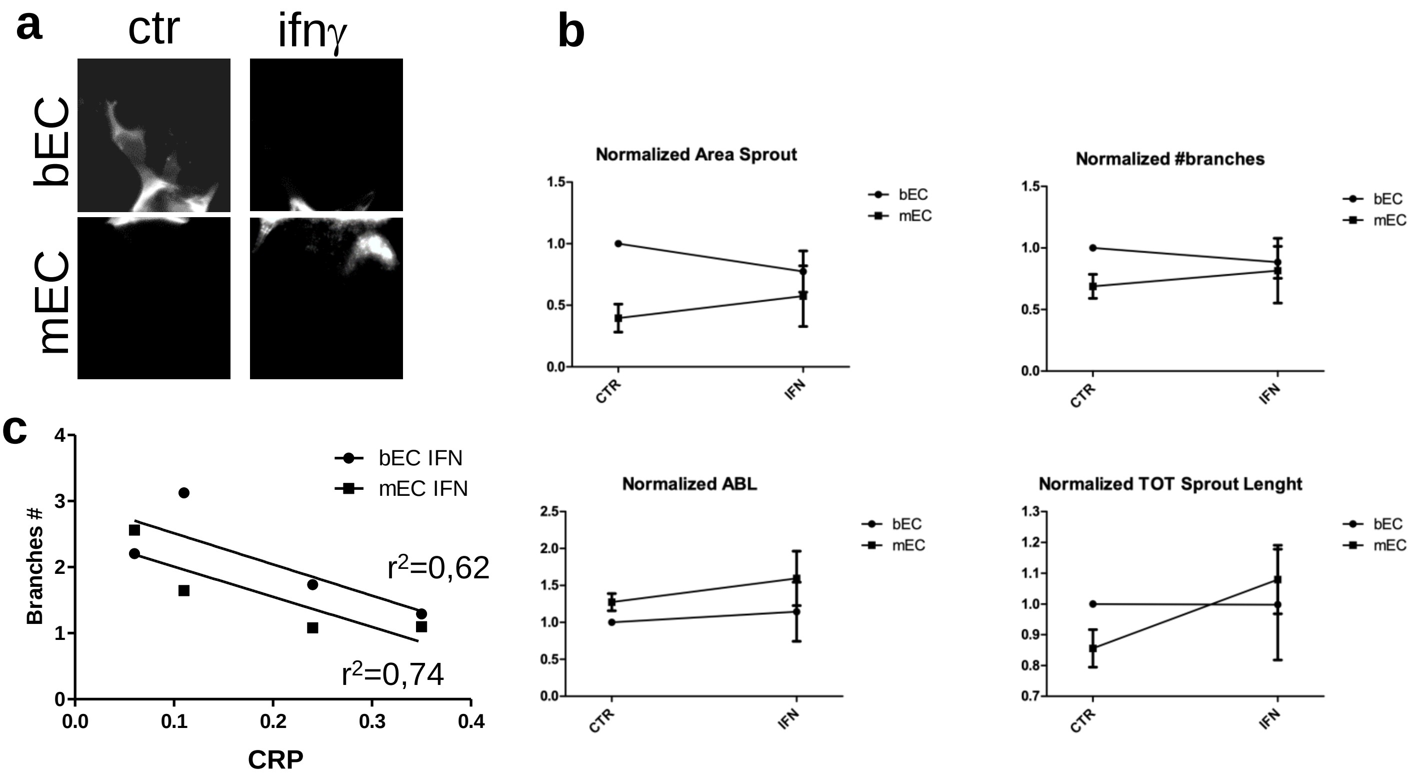

Supplementary Figure 5: Representative immunofluorescence images of bECs and mECs invading the fibrin channel in control conditions or stimulated with IFNγ. B) Graphs comparing values of branches number, area sprouting, average branch length and total sprout length for bECs and mECs in control conditions and under stimulation with IFNγ. Data were normalized on the value of bECs in control conditions, which was set at 1. Average and SEM of at least 5 replicates per patients are shown. C) Interpolation of the branch number plotted against CRP values, showing a stronger negative linear correlation in mECs (not significant, $r^2 = 0.74$) as compared to bECs ($r^2 = 0.62$), n=4.

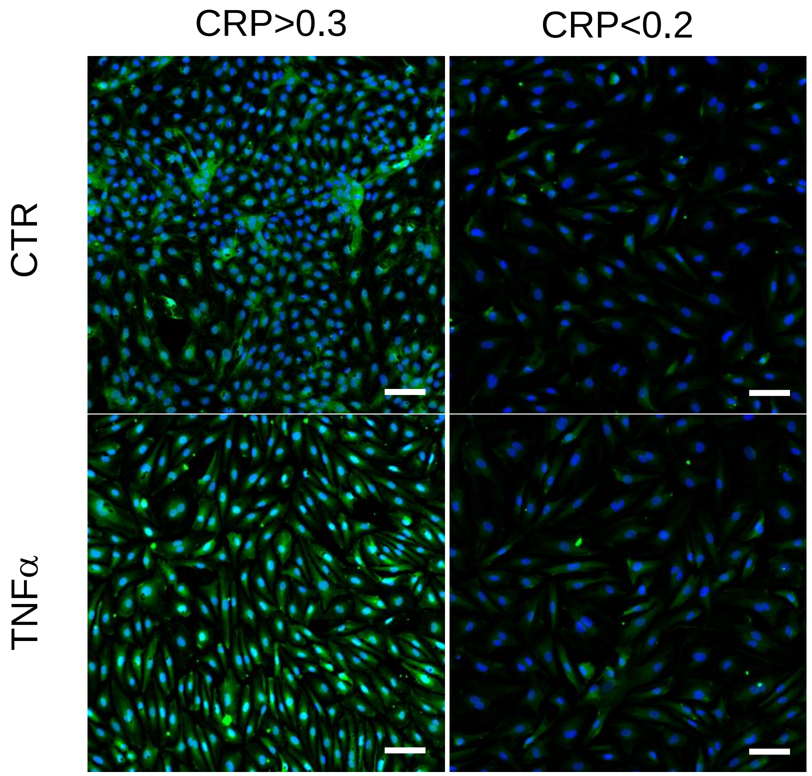

Supplementary Figure 6: Immunofluorescence images of IGFBP3 expression in cultured mECs. We compared the expression in a representative patient with high systemic inflammation value (CRP>0.3) with that in a representative patient with low systemic inflammation value (CRP<0.2). The comparison was performed in control conditions (upper row) or when stimulated with TNFα (lower row). Scale bars 50 µm.
