# Supplementary material for: Differential angiogenesis of bone and muscle endothelium in aging and inflammatory processes
Source: Commun Biol. 2023 Jan 31;6:126. doi: 10.1038/s42003-023-04515-9 (PMC9889796; doi:10.1038/s42003-023-04515-9)
Supplement: Supplementary file 3 — Reporting Summary [file 42003_2023_4515_MOESM3_ESM.pdf]

nature portfolio

Corresponding author(s): *Double-blind peer review submissions: write DBPR and your manuscript number here instead of author names.*

Last updated by author(s): *YYYY-MM-DD*

Reporting Summary

Nature Portfolio wishes to improve the reproducibility of the work that we publish. This form provides structure for consistency and transparency in reporting. For further information on Nature Portfolio policies, see our Editorial Policies and the Editorial Policy Checklist.

Statistics

For all statistical analyses, confirm that the following items are present in the figure legend, table legend, main text, or Methods section.

n/a	Confirmed	
☐	☒	The exact sample size (*n*) for each experimental group/condition, given as a discrete number and unit of measurement
☐	☒	A statement on whether measurements were taken from distinct samples or whether the same sample was measured repeatedly
☐	☒	The statistical test(s) used AND whether they are one- or two-sided<br>*Only common tests should be described solely by name; describe more complex techniques in the Methods section.*
☐	☒	A description of all covariates tested
☒	☐	A description of any assumptions or corrections, such as tests of normality and adjustment for multiple comparisons
☐	☒	A full description of the statistical parameters including central tendency (e.g. means) or other basic estimates (e.g. regression coefficient) AND variation (e.g. standard deviation) or associated estimates of uncertainty (e.g. confidence intervals)
☐	☒	For null hypothesis testing, the test statistic (e.g. *F*, *t*, *r*) with confidence intervals, effect sizes, degrees of freedom and *P* value noted<br>*Give P values as exact values whenever suitable.*
☒	☐	For Bayesian analysis, information on the choice of priors and Markov chain Monte Carlo settings
☒	☐	For hierarchical and complex designs, identification of the appropriate level for tests and full reporting of outcomes
☐	☒	Estimates of effect sizes (e.g. Cohen's *d*, Pearson's *r*), indicating how they were calculated

Our web collection on statistics for biologists contains articles on many of the points above.

Software and code

Policy information about availability of computer code

Data collection	Feature Extraction software v10.7.3.1 (Agilent Technologies) for the acquisition of images from gene microarrays
Data analysis	For analysis of transcroptomic data: Bioconductor (www.bioconductor.org), with LIMMA (Linear Models for Microarray Analysis) R package<br>For quantification of angiogenesis parameters: Image J (Fiji) version 1.51<br>For statistical analysis: Graphad PRISM version 8<br>For computational simulations: Comsol Multiphysics

For manuscripts utilizing custom algorithms or software that are central to the research but not yet described in published literature, software must be made available to editors and reviewers. We strongly encourage code deposition in a community repository (e.g. GitHub). See the Nature Portfolio guidelines for submitting code & software for further information.

Data

Policy information about availability of data

All manuscripts must include a data availability statement. This statement should provide the following information, where applicable:

- Accession codes, unique identifiers, or web links for publicly available datasets
- A description of any restrictions on data availability
- For clinical datasets or third party data, please ensure that the statement adheres to our policy

Raw and processed transcriptomic data were deposited on the GEO Omnibus database (GSE172419).

Human research participants

Policy information about studies involving human research participants and Sex and Gender in Research.

Reporting on sex and gender	We enrolled 31 osteoarthritic patients, 13 males and 18 females, and successful isolation of both bone and muscle ECs was achieved for 17 patients, 7 males and 10 females. We did not analyze our results in funtion of patient sex, since it was not a factor possibly linked to the parameters involved in our study (i.e. OA degree and more generally inflammation)
Population characteristics	We collected information about age, which ranged from 43 to 76 years, BMI (22.1-36.7), OA grade measured with Tonnis score (2-3), systemic inflammation values (CRP: 0.02-0.45 and ESR 2-29). Values were typical for end stage OA patients.
Recruitment	Patients were recruited among end stage OA patients, who underwent a hip prostehsis implantation. During the pre-surgical visit, patients were asked to participate, and signed an informed consent. SInce we could recruit only patients selected for surgery, a limitation in our study is thus that we could not analyze patients with early OA
Ethics oversight	Hospital san Raffaele Ethical Committee

Note that full information on the approval of the study protocol must also be provided in the manuscript.

Field-specific reporting

Please select the one below that is the best fit for your research. If you are not sure, read the appropriate sections before making your selection.

☒ Life sciences ☐ Behavioural & social sciences ☐ Ecological, evolutionary & environmental sciences

For a reference copy of the document with all sections, see nature.com/documents/nr-reporting-summary-flat.pdf

Life sciences study design

All studies must disclose on these points even when the disclosure is negative.

Sample size	We did not estimate the number of patient needed before starting the study, since we could not quantify the possible effects of the variable (endothelial origin) that we wanted to assess. We recruited all the possible patients in a 2 years period, optimizing the isolation protocol to maximise the number of samples achievable
Data exclusions	No data were excluded, some patients were not analyzable because we could not isolate cells from their waste surgical pieces
Replication	For some of the patients, we repeated the analysis on angiogenic behavior on a further group of microfluidic chips. Values of angiogenic sprouting measured on the new group were in the range of previously measured chips.
Randomization	n/a
Blinding	n/a

Reporting for specific materials, systems and methods

We require information from authors about some types of materials, experimental systems and methods used in many studies. Here, indicate whether each material, system or method listed is relevant to your study. If you are not sure if a list item applies to your research, read the appropriate section before selecting a response.

Materials & experimental systems

n/a	Involved in the study	
☐	☒	Antibodies
☒	☐	Eukaryotic cell lines
☒	☐	Palaeontology and archaeology
☒	☐	Animals and other organisms
☐	☒	Clinical data
☒	☐	Dual use research of concern

Methods

n/a	Involved in the study	
☒	☐	ChIP-seq
☒	☐	Flow cytometry
☒	☐	MRI-based neuroimaging

Antibodies

Antibodies used	mouse monoclonal anti CD144, Thermofisher, 16B1, # 12-1449-80<br>mouse monoclonal anti-rat ICAM-1, Thermofisher, 1A29, #MA5407<br>rabbit polyclonal anti human IGFBP3, Thermofisher, #PA5-27190<br>rabbit polyclonal anti-NOSTRIN Thermofisher, #PA5-113121<br>rabbit polyclonal anti-human CNGL-1, Thermofisher, #PA5-55661<br>mouse monoclonal anti CD62E, Thermofisher, P2H3, #14-0627-82<br>rabbit polyclonal anti-human SULF-1, Thermofisher, #PA5-115984<br>rabbit polyclonal anti human DcR3, Thermofisher, #PA5-86474<br>mouse monoclonal antiOPG, Thermofisher, 9C1, #MA5-34922<br>recombinant anti CD31 (biotin), AbCam, EPR3094, ab199734<br>Biotinylated Ulex Europaeus Agglutinin I, Vector Labs, #: B-1065-2
Validation	All antibodies were selected for reactivity against human and with verification of compatibility with immunohistochemistry on frozen and paraffin embedded samples

Clinical data

Policy information about clinical studies

All manuscripts should comply with the ICMJE guidelines for publication of clinical research and a completed CONSORT checklist must be included with all submissions.

Clinical trial registration	NCT04047459
Study protocol	Clinicaltrial.gov
Data collection	Patients recruited from 6/6/2016 to 31/05/2018, data analysis afterwards
Outcomes	The study was originally designed to find differences between bone and muscle endothelial cells that could explain the different extravasation of cancer cells. Analyzing the transcriptomic results however it resulted evident how differences could be influenced by the underlying OA pathology, so we modified the initially forseen primary and secondary outcomes of the study
